# Superior electro-optic response in multiferroic bismuth ferrite nanoparticle doped nematic liquid crystal device

**DOI:** 10.1038/srep10845

**Published:** 2015-06-04

**Authors:** Prasenjit Nayek, Guoqiang Li

**Affiliations:** 1 Department of Ophthalmology and Visual Science, The Ohio State University, 1330 Kinnear Road, Columbus, Ohio 43212; 2 Department of Electrical and Computer Engineering, The Ohio State University, 1330 Kinnear Road, Columbus, Ohio 43212.

## Abstract

A superior electro-optic (E-O) response has been achieved when multiferroic bismuth ferrite (BiFeO_3_/BFO) nanoparticles (NPs) were doped in nematic liquid crystal (NLC) host E7 and the LC device was addressed in the large signal regime by an amplitude modulated square wave signal at the frequency of 100 Hz. The optimized concentration of BFO is 0.15 wt%, and the corresponding total optical response time (rise time + decay time) for a 5 μm-thick cell is 2.5 ms for ~7 V_rms_. This might be exploited for the construction of adaptive lenses, modulators, displays, and other E-O devices. The possible reason behind the fast response time could be the visco-elastic constant and restoring force imparted by the locally ordered LCs induced by the multiferroic nanoparticles (MNPs). Polarized optical microscopic textural observation shows that the macroscopic dislocation-free excellent contrast have significant impact on improving the image quality and performance of the devices.

A fast response time is a challenging task for liquid crystals (LCs) electro-optic devices. Apart from display applications^1^, LCs play dominant role in biomedical optical devices like switchable electro-optic diffractive lenses for ophthalmic applications^2^,^3^,^4^,^5^, phase-only spatial light modulators (SLMs) for advanced microscopy^6^, creation of complex diffraction patterns for optical trapping^7^,^8^, and high-energy near-infrared laser applications^9^, tunable filters^6^, and integrated optics^10^, etc. For all the above mentioned applications, one of the basic requirement is faster response time. Faster response time is also vital for next generation three-dimensional (3D) LC display devices (LCDs) in order to reduce motion blur, obtain a field sequential color and overcome crosstalk^11^,^12^,^13^,^14^. For LCDs, the targeted 3 ms response time is optimal for reducing motion blurs and color break up. Different techniques for improving the response time have been attempted, e.g., using low viscosity materials^15^, pixel design, electrode shape, driving scheme^16^, thin cell gap^17^, anchoring energy^18^, overdrive schemes^19^,^20^, guest-host materials^21^, new switching modes^22^,^23^, and incorporating nematic LCs into the polymer matrices^24^,^25^, but improvements are limited. Although Borshch *et al.*^26^ demonstrated a response time of about 30 ns for both the field-on and field-off switching by the electric field induced modification of the order parameters, its implementation is not simple. It requires high voltage (up to 1 kV with nanosecond rise and fall fronts) and it needs two additional prisms, which may not be convenient for practical applications. Recently, blue phase mode LCDs show faster response times but still need to overcome hysteresis, high operating voltages, and narrow temperature ranges for wide spread applications^27^. Another important technique for achieving the faster response time is through control of the long range orientational order of the LC^28^,^29^,^30^,^31^. Dislocation defect-free orientation control is one of the fundamental demands for the preparation of such LC based devices. Nanoparticle–LC mixtures are fascinating for topological defect induction and self-assembled long range orientation order structure through elastic distortions in the LC host^32^,^33^,^34^,^35^,^36^,^37^. Theoretical predictions have been made that nanoparticle liquid-crystal mediated long range interactions could be controlled by changing the anchoring energy and the particle diameter^38^. Kobayashi *et al.* contributed a lot for different metal NPs (palladium, silver) doped in LC and explained the results^39^,^40^,^41^. Ferroelectric LC doped with silica NPs which do not have either ferrroelectricity or ferrromagneticity has been studied^42^. Recently ferroelectric nanoparticle (FNP)-NLC mixers demonstrated strong electro-optic response in low refractive index material^43^. In LC switching, FNPs have made a significant impact on E-O performance and demonstrated reduction of Freédericksz threshold voltage, decrease in the switching time (τ_on_), and increase in the restoring switching time (τ_off_)^44^. Ferroelectric thio-hypo-diphosphate (Sn_2_P_2_S_6_) doped in NLC host was studied by Ouskova *et al.*^45^ and observed a strong influence of the dielectric absorption spectrum of the molecular rotation around the short axis. BiFeO_3_ is a unique material that simultaneously exhibits ferroelectric (Curie temperature (T_C_) = 830 °C) and long-range antiferromagnetic G-type (Neel temperature (T_N_) = 370 °C) ordering^46^,^47^ which have superior electric and magnetic properties^48^. BFO NP-NLC system may play a better role due to its strong room temperature ferroelectric polarization which occurs along the pseudocubic (111) direction with a magnitude of 90–95 μC/cm^2^ which is remarkable with respect to other ferroelectric material^49^. It has potential possibility to perturb LC ordering by polarization and/or magnetization and the subsequent effect on LC performances. In this work we have synthesized the BFO nanoparticles and studied the E-O properties of BFO NP–NLC (E7) mixtures and demonstrated excellent results. We have achieved some promising response time which could be exploited in adaptive optical devices, display devices and other photonic devices where faster E-O performance is needed.

## Experiments

The LC used was the eutectic LC mixture, commercially known as E7 (Merck). The abbreviation E7 stands for a LC mixture consisting of several types of cyanobiphenyls, mainly 5CB and in less quantity, triphenyl. E7exhibits a nematic phase in the temperature interval from −10 °C up to the transition to the isotropic phase at T_NI_ = 58 °C. E7 has a birefringence, Δn = 0.2253 (at 20 °C and λ = 589.3 nm), dielectric anisotropy, Δε = 13.89 (positive anisotropy), and a rotational viscosity, γ = 166 mPa s. Polyimide coated ITO substrates have complementary anti-parallel alignment to diminish performance degradation from non-uniformity. BiFeO3 nano-powder was synthesized by the conventional sol-gel route. Bismuth nitrate ((BiNO_3_)_3_.5H_2_O) and iron nitrate ((FeNO_3_)_3_.9H_2_O) were used as precursor materials. All precursor compounds were in analytical grade purity and used as received from Sigma Aldrich without further purification. Stoichiometric amount of 0.05 molar bismuth nitrate and iron nitrate were prepared by adding the proper amount of precursor to 25 ml diluted nitric acid solvent. These two solutions were mixed and stirred at room temperature for 30 minutes, then 10 mL of 0.1 molar aqueous citric acid solution was added as gelatin material to the foresaid mixture. The final mixture was stirred with a magnetic stirrer and kept at 80 °C to dry. The brown colored dried gel is then annealed at 650 °C (heating rate 5 °C/min.) for 3 hours. This annealed powder is used for various characterizations. Three different mixtures were prepared with BFO content 0.15 wt%, 0.58 wt%, and 1 wt% respectively mixed in host E7. Mixtures were sonicated for 40 minutes and then stirred at 70 °C for 4-5 hours. The ITO coated planar aligned (anti-parallel rubbed polyimide layer) cell, with an effective area of 5 mm^2^ and a cell gap of 5 μm was thermally treated at 70 °C for 10 minutes before the mixtures were injected into the cell. The BFO-NLC mixtures were injected into the cell. The filled cells were kept at 70 °C for one hour and then cooled down to room temperature. X-Ray diffraction spectrum was taken with Rigaku SmartLab high resolution diffractometer. The morphologies of the BFO NPs were characterized by Zeiss Ultra 55 plus field emission scanning electron microscope (FESEM). The textures were taken using a polarizing optical microscope (POM) Leitz Laborlux 12 Pol, which is fitted with a digital camera (Nikon). The electro-optic response was measured by placing the cells between a pair of crossed polarizers, and maintaining the rubbing direction of the cell at 45° angle to the transmission axes of the polarizers. The input voltage of the cell was applied by a function generator (Agilent 33220A). The amplitude modulated square wave (10 V_p_), with a frequency of 100 Hz (carrier frequency 1 k Hz) was applied to the LC cell and the output was detected by a photodiode detector (818-BB-20 from Newport Corporation). The output signal from the detector is fed into the digital storage oscilloscope DS03202A (Agilent), which was interfaced with a computer. The wavelength of the He-Ne laser light was 544 nm. The transmission vs voltage was measured by power meter (NOVA II) and recorded digitally by data acquisition system (cDAQ-9172) using LabView software.

## Results and Discussion

**Figure 1:**
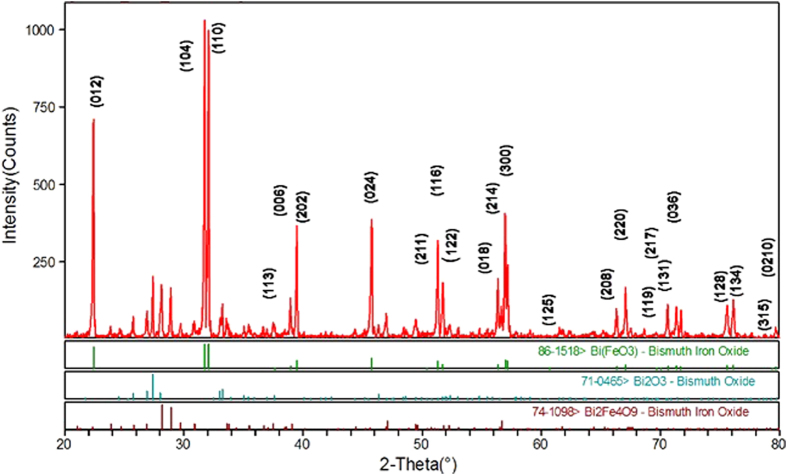
X-ray diffraction pattern for the synthesized BFO.

**Figure 2:**
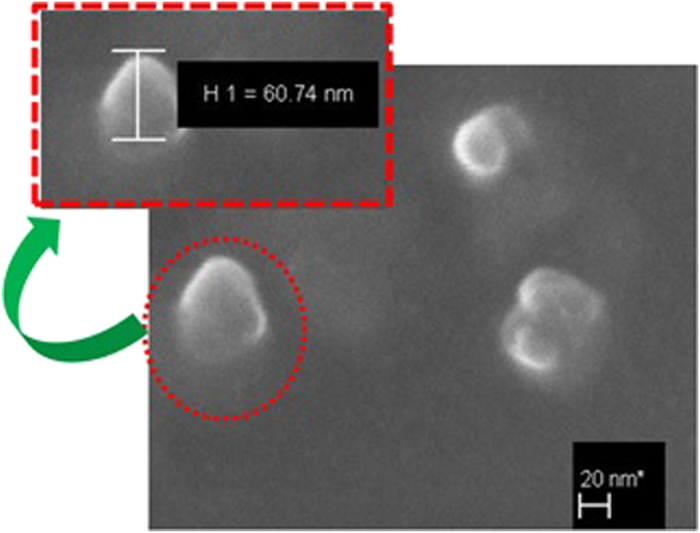
FESEM image of the synthesized BFO.

**Figure 3:**
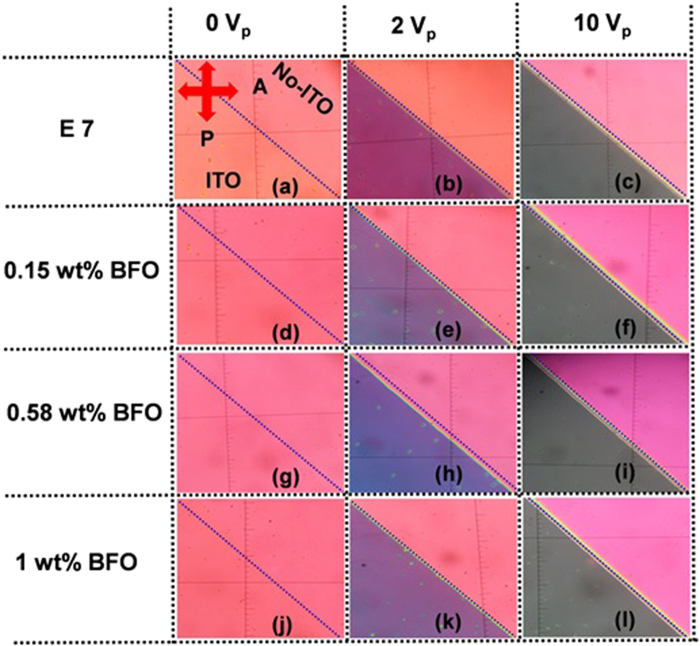
POM images of E7 and E7 + BFO mixtures with different concentration (wt%) across the regions with and without ITO. (**a**), (**b**), and (**c**), pristine E7 at 0 V, 2 V and 10 V_p_ respectively; (**d**), (**e**), and (**f**), E7 + 0.15 wt% BFO-NP mixture at 0 V, 2 V and 10 V_p_, respectively; (**g**), (**h**), and (**i**), E7 + 0.58 wt% BFO-NP mixture at 0 V, 2 V and 10 V_p_, respectively; and (**j**), (**k**), and (**l**), E7 + 1 wt% BFO-NP mixture at 0 V, 2 V and 10 V_p_, respectively.

**Figure 4:**
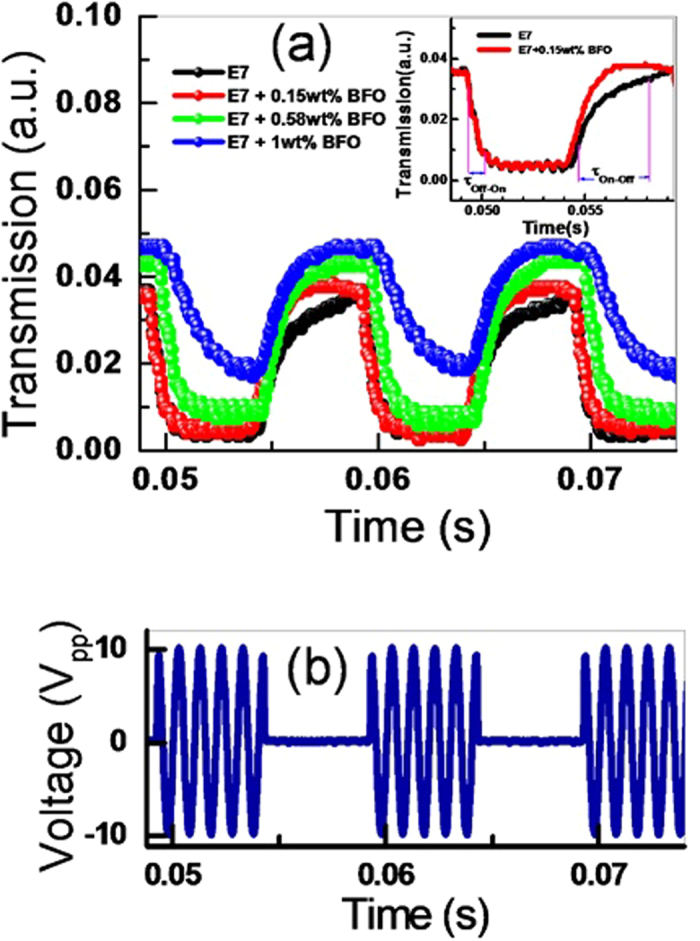
(a) Transmission vs time for E7 and different mixtures. The inset picture shows the responses of E7 and the best performed mixture of 0.15 wt% NPs and E7; (**b**) The input voltage vs time that was used to measure the response time of E7 and the mixtures. A 10 V_p_ and 100 Hz amplitude modulated square wave signal (carrier frequency 1 k Hz) was used.

**Figure 5:**
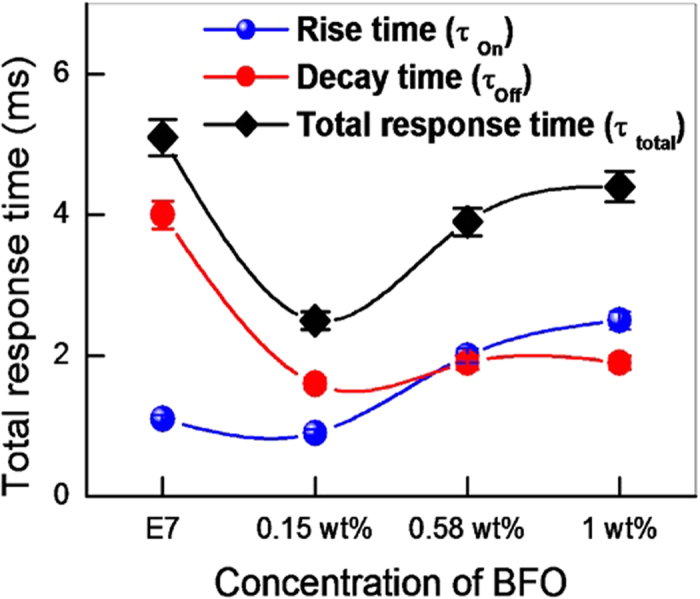
The rise time (*τ*_*on*_), decay time (*τ*_*off*_), and total response time (*τ*_*total*_ = *τ*_*on*_ + *τ*_*off*_). During the Off-On and On-Off time convention for time difference T_10%_ - T_90%_ and T_90%_ - T_10%_ have followed and the total response time for the pristine and composite material. The total response time for all the three mixtures gets faster by 14%, 24% and 51% for 1 wt% BFO, 0.58 wt% BFO and 0.15 wt% BFO respectively compared to pristine NLC E7.

**Figure 6:**
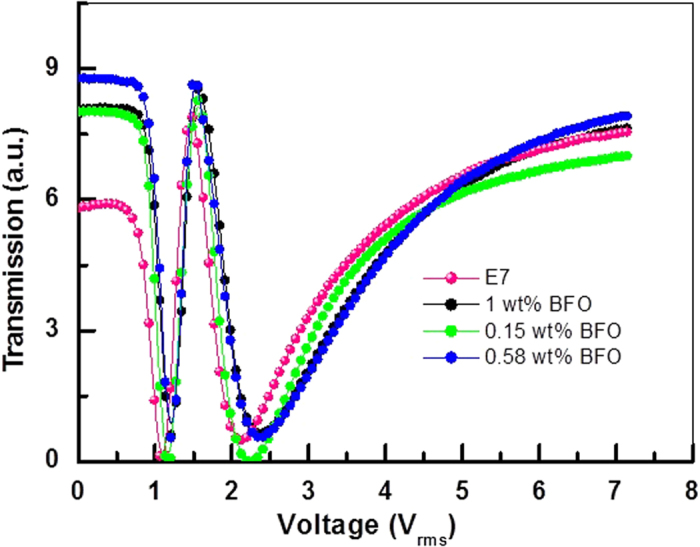
Transmission vs voltage (V-T) graph for all the devices. The transmission is taken when the polarizer and analyser are parallel to each other. Measured threshold voltage for E7, 0.15 wt% BFO, E7 + 0.58 wt% BFO, E7 + 1 wt% BFO are 0.77 V, 0.85 V, 0.9 V and 0.9 V respectively.

**Figure 7:**
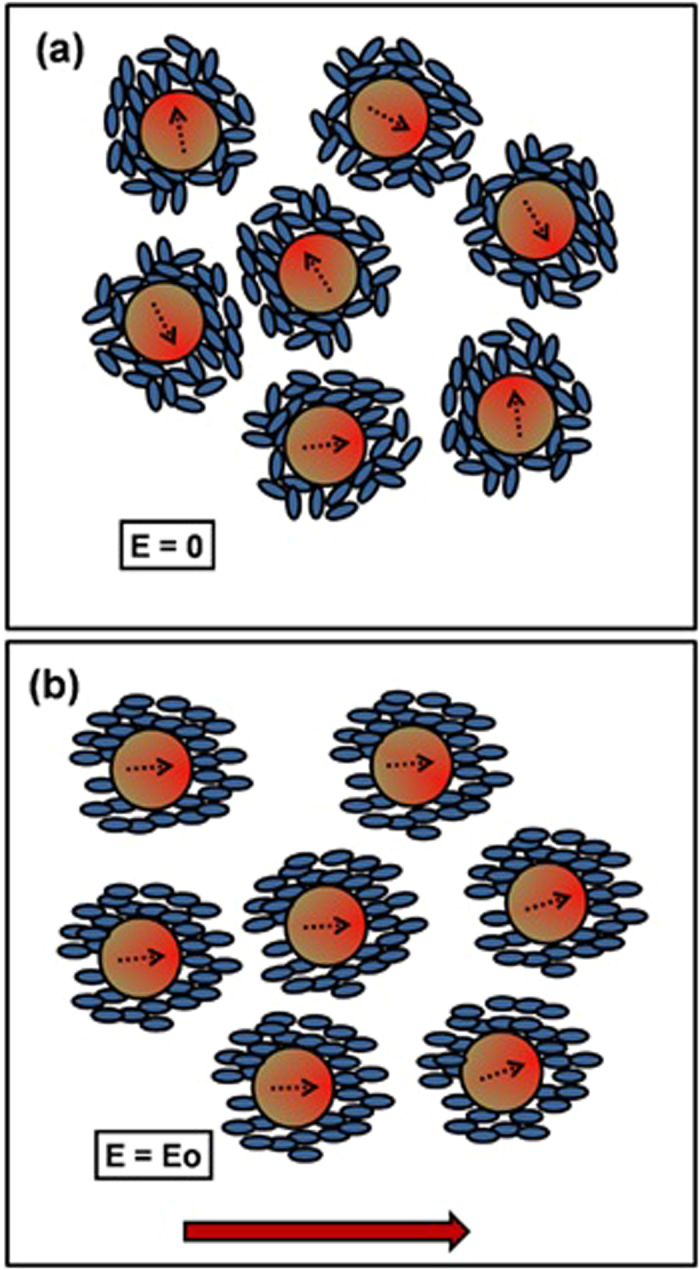
The schematic diagram of the local ordering surrounding the nanoparticles (a) without electric field, (b) with electric field. (The brown arrows indicate the direction of the applied electric field). We have focused only on the sites near the NPs. Circles with black arrows are for the NPs. Direction of polarization of the ferroelectric NPs has been shown by the arrow.

Figure 1 shows the X-ray diffraction pattern and XRD data that were collected using Cu Kα radiation generated at 40 kV and 44 mA. The diffraction angle (2θ) was varied, 20° ≤ 2θ ≤ 80°, with a 0.02° step size (Δ2θ). The reflection peaks of the sample prepared can be indexed as a single-phase perovskite structure belonging to the space group R3c, which is in good agreement with the literature data of JCPDS Card No. 86-1518^50^. The calculated lattice parameters (a = b = 5.5774 Å and c = 13.8616 Å) are in agreement with rhombohedral BFO nanoparticles according to the reported data (lattices in three dimensions generally have three lattice constants, referred to as *a*, *b*, and *c*)^51^. There are also traces for Bi_2_Fe_4_O_9_(JCPDS Card No. 74-1098) and Bi_2_O_3_(JCPDS Card No. 71-0465) particles. The average crystallite size was obtained from the Debye–Scherrer formula^52^:
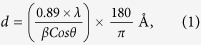
where d is the crystallite size, λ is the wavelength (1.54056 Å) of Cu Kα radiation, β is the full width at half maxima (FWHM = 0.13016), θ is the diffraction angle (15.857 degrees). The average crystallite size obtained from the Eq. (1) was 62.74 nm. The FESEM picture is depicted in Fig. 2 and it reveals the shape and size of the BFO NPs. Figure 3 shows the POM images of E7 and E7 + BFO mixtures with different concentrations (wt%) across the regions with and without ITO. The texture shows that there are no significant defects and have very good contrast at 10 V_p_ indicating that it can be exploited for display devices as well^53^,^54^. The pictures were taken under the microscope by fixing the polarizer and analyzer whose transmission axes are at 90° to each other with the smallest division of the scale at 20 μm. The cell gap of all the samples was 5 μm. The rubbing direction was 45° with respect to the transmission axes of the polarizer and the analyzer. We placed the cell in such a way that half of the texture shows the ITO coated portion whereas the other half is the non ITO coated portion in order to compare the relative textural change under the electric field addressing with respect to the non-addressing portion. A function generator was used to apply the electric field in the form of a sine wave with amplitude of 10 V_p_ and a frequency of 1 kHz. The ITO coated portion of the cell changes color due to voltage induced reorientation of LCs. At 10 V_p_, the ITO coated portion gets black which indicates the LCs are in homeotropic configuration. We have not observed any significant defect in the texture due to the nanoparticle aggregates and the contrast is good. The electro-optic responses of the pristine E7 NLC and the other mixtures are shown in Fig. 4(a) with the input voltage shown in Fig. 4(b). Figure 5 shows the On-Off response times (*τ*_*off*_, time calculated between 90% and 10% of the transmittance level) for E7, E7 + 0.15 wt% BFO, E7 + 0.58 wt% BFO and E7 + 1 wt% BFO are 4 ms, 1.6 ms, 1.9 ms, and 1.9 ms respectively, whereas the Off-On response times (*τ*_*on*_, time calculated between 10% and 90% of the transmittance level) for E7, E7 + 0.15 wt% BFO, E7 + 0.58 wt% BFO and E7 + 1 wt% BFO are 1.1 ms, 0.9 ms, 2 ms, and 2.5 ms respectively. It is evident from the inset picture of Fig. 4(a) that for E7, the field “On-Off” response time is longer and the “Off-On” response time is shorter. For 0.15 wt% BFO doped system, the “Off-On” response time is the shortest. The total response time (τ_On_ + τ_Off_) for all the three mixtures gets faster by ~51% , ~24%, and ~14%, for E7 + 0.15 wt% BFO, E7 + 0.58 wt% BFO, and E7 + 1 wt% BFO respectively compared to the host E7. The pure NLC rise time (*τ*_*on*_) can be defined as^26^:
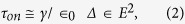
where *γ* is rotational viscosity, Δ∈ is the dielectric anisotropy of the LC, E is the electric field, and ∈_0_ is the dielectric permittivity in free space. The decay (or switching off) time (*τ*_*off*_) can be written as^26^:
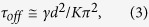
where d is the cell thickness and K is the elastic constant. Figure 6 depicts that the threshold voltage does not change significantly (within 1 V for all the cells), so we can reasonably assume the elastic constant doesn’t change significantly as the threshold voltage holds for equation^55^:
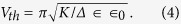


Equation (3) explicitly depends on the viscosity as the other parameters remain fixed. Apparently the determining factor for the improvement of the decay time should be the decrease in the viscosity, but the rise time should also decrease simultaneously for all the mixtures which is opposed to our experimental results. The experimental results show that, only for lower concentration of BFO (0.15 wt%), there is a decrease in both the rise time and the decay time. Whereas for the other mixtures, the rise time increases but the decay time decreases in comparison with the host E7. For low concentrations (0.15 wt%) of BFO, lower viscosity may play a partial role for the fastening of the rise time but not for higher concentrations of BFO. In lower concentration systems, a decrease in viscosity may cause the decrease in viscoelastic constant (*γ*/*K*) due to coupling of strong ferroelectric polarization of NPs to LCs over dipole-dipole coupling in LCs. The calculated (from Eq. (3)) viscoelastic constant for the four cases are as follows: (γ/K)_E7_ = 1.58 ms/μm^2^, (γ/K)_E7 + 0.15wt% BFO_ = 0.65 ms/μm^2^, (γ/K)_E7 + 0.58wt% BFO_ = 0.75 ms/μm^2^, and (γ/K)_E7 + 1wt% BFO_ = 0.75 ms/μm^2^. When the concentration of the NPs increases, the viscoelastic coefficient and rise time increases but the decay time has not increased as expected from above equations. In addition, the threshold voltage almost remains fixed. The threshold voltage should also decrease due to the ferroelectric effect, so there might be some other possible reason for those abnormalities. We are proposing this might be due to the origin of a restoring force by the locally oriented LC regions by the NPs when the field is turned “Off.” A schematic diagram is demonstrated in Fig. 7 for clarification, where the NPs are represented by reddish circles and the liquid crystals are represented by bluish ellipses. When no electric field is applied, the liquid crystals nearer to the NPs could orient under the influence of the NPs’ spontaneous polarization electric field with respect to the LCs far from the NPs^56^. If we consider a NP a dipole, then the local electric field due to this dipole can be written as:
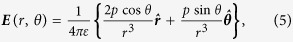
where p is the dipole moment, ***râ*** is the position vector, *θ* is the polar angle, and *ε* is the dielectric permittivity of the medium. With reference to the format 
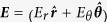
, the amplitude of the electrical field can be written as
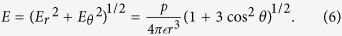


If we consider the radius of the NP is a and the dipole moment of the sphere is 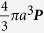, where ***P*** is the polarization of the NP, then
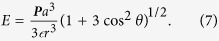


We have neglected the higher order correction term for the anisotropy of LC. Also there might have some anchoring force between the NPs and the LCs. This interaction may create some local randomly oriented regions^57^. Due to this random nature of the locally oriented structures, the external electric field will expend some energy to perturb those constrained regions along the electric field. For this reason, the threshold voltage was not decreased for the mixtures. The LCs outside the influence of the locally aligned region also orient along the electric field. Under sufficient electric field, the LCs close to the local regions as well as other NLCs orient along the electric field. When the electric field goes from the “On” to “Off” state, the local regions tend to return to their previously aligned positions, so there will be a stronger restoring force which can fasten the response of the device. It is important to note that the “Off-On” response time does not change significantly for 0.15 wt% BFO doped device, but the “On-Off” response time improves excellently due to this additional restoring force imparted by the MNPs-induced local regions.

## Conclusion

In conclusion, we have studied a unique system where faster response time results from the combined effects of the viscosity and the restoring force imparted by the locally ordered LCs which are induced by the MNPs. This restoring force helps to reduce the decay time of the device. It reveals that for low concentration of MNPs, doping is essential for achieving macroscopic dislocation-free fast-response electro-optic devices. For higher concentration of the MNPs, this restoring force slowed down the response due to higher viscosity.

## Additional Information

**How to cite this article**: Nayek, P. and Li, G. Superior electro-optic response in multiferroic bismuth ferrite nanoparticle doped nematic liquid crystal device. *Sci. Rep.* **5**, 10845; doi: 10.1038/srep10845 (2015).
